# 1′-Methyl-4′-[4-(trifluoro­meth­yl)phen­yl]dispiro­[acenaphthyl­ene-1,2′-pyrrolidine-3′,2′′-indane]-2,1′′(1*H*)-dione

**DOI:** 10.1107/S1600536812013645

**Published:** 2012-04-04

**Authors:** Ang Chee Wei, Mohamed Ashraf Ali, Tan Soo Choon, Suhana Arshad, Ibrahim Abdul Razak

**Affiliations:** a Institute for Research in Molecular Medicine, Universiti Sains Malaysia, Minden 11800, Penang, Malaysia; b School of Physics, Universiti Sains Malaysia, 11800 USM, Penang, Malaysia

## Abstract

In the title compound, C_31_H_22_F_3_NO_2_, the pyrrolidine and cyclo­pentane rings within the dihydro­indene ring system are in envelope conformations, with the N atom and the spiro-C atom at the flap, respectively. An intra­molecular C—H⋯O hydrogen bond forms an *S*(8) ring motif. The mean plane through the pyrrolidine ring [r.m.s. deviation = 0.179 (2) Å] makes dihedral angles of 86.30 (13), 88.99 (10) and 79.69 (11)° with the benzene ring, the dihydro­acenaphthyl­ene ring and the mean plane of the indane system, respectively. In the crystal, mol­ecules are linked by C—H⋯O and C—H⋯N hydrogen bonds into a two-dimensional network parallel to the *ac* plane. C—H⋯π inter­actions further stabilize the crystal structure.

## Related literature

For the structures of related heterocyclic compounds with anti­tubercular activity, see: Wei, Ali, Choon *et al.* (2011 ▶, 2012 ▶); Wei, Ali, Ismail *et al.* (2011 ▶). For ring conformations, see: Cremer & Pople (1975 ▶). For hydrogen-bond motifs, see: Bernstein *et al.* (1995 ▶). For the stability of the temperature controller used in the data collection, see: Cosier & Glazer (1986 ▶).
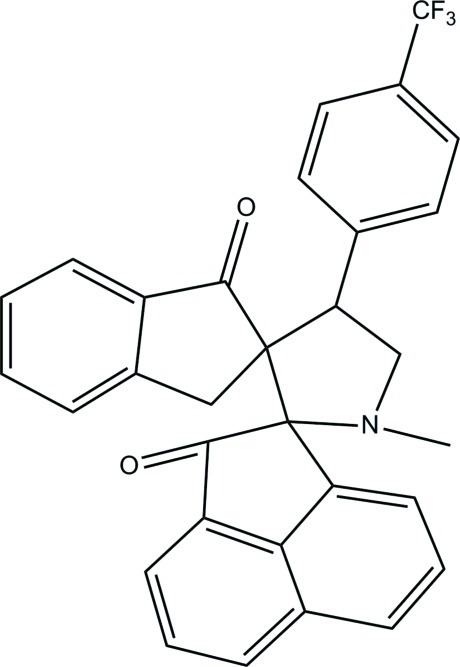


## Experimental

### 

#### Crystal data

C_31_H_22_F_3_NO_2_
*M* *_r_* = 497.50
Monoclinic, 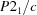

*a* = 8.8373 (2) Å
*b* = 20.1333 (5) Å
*c* = 13.7129 (3) Å
β = 96.243 (1)°
*V* = 2425.39 (10) Å^3^
*Z* = 4
Mo *K*α radiation
μ = 0.10 mm^−1^
*T* = 100 K
0.30 × 0.28 × 0.20 mm

#### Data collection

Bruker SMART APEXII CCD area-detector diffractometer
Absorption correction: multi-scan (*SADABS*; Bruker, 2009 ▶) *T*_min_ = 0.971, *T*_max_ = 0.980
27133 measured reflections
7039 independent reflections
4753 reflections with *I* > 2σ(*I*)
*R*_int_ = 0.072

#### Refinement

*R*[*F* ^2^ > 2σ(*F* ^2^)] = 0.082
*wR*(*F* ^2^) = 0.186
*S* = 1.10
7039 reflections
335 parameters
H-atom parameters constrained
Δρ_max_ = 0.55 e Å^−3^
Δρ_min_ = −0.37 e Å^−3^

### 

Data collection: *APEX2* (Bruker, 2009 ▶); cell refinement: *SAINT* (Bruker, 2009 ▶); data reduction: *SAINT*; program(s) used to solve structure: *SHELXTL* (Sheldrick, 2008 ▶); program(s) used to refine structure: *SHELXTL*; molecular graphics: *SHELXTL*; software used to prepare material for publication: *SHELXTL* and *PLATON* (Spek, 2009 ▶).

## Supplementary Material

Crystal structure: contains datablock(s) global, I. DOI: 10.1107/S1600536812013645/rz2729sup1.cif

Structure factors: contains datablock(s) I. DOI: 10.1107/S1600536812013645/rz2729Isup2.hkl

Additional supplementary materials:  crystallographic information; 3D view; checkCIF report

## Figures and Tables

**Table 1:** Hydrogen-bond geometry (Å, °) *Cg*1 is the centroid of the C15–C20 ring.

*D*—H⋯*A*	*D*—H	H⋯*A*	*D*⋯*A*	*D*—H⋯*A*
C29—H29*A*⋯O1	0.95	2.29	3.166 (3)	153
C4—H4*A*⋯O2^i^	0.95	2.52	3.364 (3)	147
C16—H16*A*⋯N1^ii^	0.95	2.51	3.429 (3)	163
C26—H26*A*⋯O1^iii^	0.95	2.51	3.324 (3)	144
C5—H5*A*⋯*Cg*1^iv^	0.95	2.74	3.417 (3)	129

Symmetry codes: (i) 
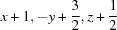
; (ii) 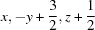; (iii) 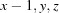; (iv) 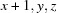.

**Fig. 1.**
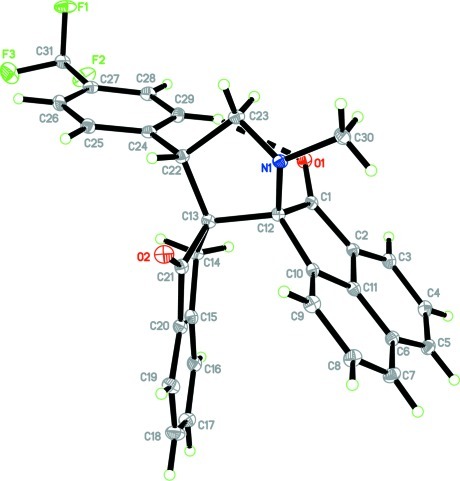
The molecular structure of the title compound, showing 30% probability displacement ellipsoids.

**Fig. 2.**
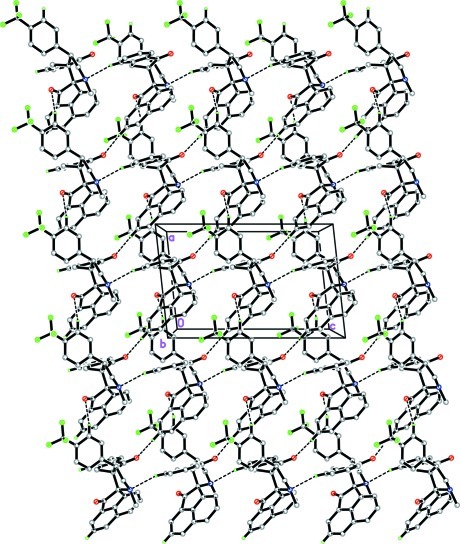
The crystal packing of the title compound. H atoms not involved in intermolecular interactions (dashed lines) have been omitted for clarity.

## supplementary crystallographic information

### Comment

As part of our ongoing search to discover novel heterocyclic compounds with antitubercular activity (Wei, Ali, Choon *et al.*, 2012; Wei, Ali, Ismail *et al.*, 2011), our group has synthesized the title compound as described below.

The molecular structure is shown in Fig. 1. The bond lengths and angles are within normal ranges and comparable to those found in related structures (Wei, Ali, Choon *et al.*, 2012; Wei, Ali, Ismail *et al.*, 2011; Wei, Ali, Choon *et al.*, 2011). The pyrrolidine ring (N1/C12/C13/C22/C23) and the cyclopentane ring (C13–C15/C20/C21) within the dihydroindene moiety are in envelope conformations, with puckering parameters (Cremer & Pople, 1975) *Q* = 0.403 (2) Å and φ= 3.9 (4)° with atom N1 at the flap, and *Q* = 0.246 (3) Å and φ= 7.8 (6)° with atom C13 at the flap, respectively. An intramolecular C29—H29A···O1 hydrogen bond (Table 1) forms an *S*(8) ring motif (Bernstein *et al.*, 1995). The dihedral angles between the mean plane through the pyrrolidine ring (N1/C12/C13/C22/C23) [r.m.s deviation of 0.179 (2) Å] with the benzene ring (C24–C29), the dihydroacenaphthylene ring (C1–C10/C12) and the mean plane of the dihydroindene ring (C13–C21) are 86.30 (13), 88.99 (10) and 79.69 (11)°, respectively.

In the crystal packing (Fig. 2), the molecules are linked into two-dimensional layers parallel to *ac* plane *via* intermolecular C4—H4A···O2, C16—H16A···N1 and C26—H26A···O1 (Table 1) hydrogen bonds. The crystal structure are further stabilized by intermolecular C5—H5A···*Cg*1 (Table 1) interactions (*Cg*1 is the centroid of the C15–C20 ring).

### Experimental

A mixture of (*E*)-2-[4-(trifluoromethyl)benzylidene]-2,3-dihydro-1*H*-indene-1-one (0.001 mol), acenaphthenequinone (0.001 mol) and sarcosine (0.002 mol) were dissolved in methanol (10 ml) and refluxed for 4 h. After completion of the reaction as evident from TLC, the excess solvent was evaporated slowly and the product was separated and recrystallized from methanol to give the title compound as yellow crystals.

### Refinement

All H atoms were positioned geometrically [C–H = 0.95 and 1.00 Å] and refined using a riding model with *U*_iso_(H) = 1.2 or 1.5*U*_eq_(C). A rotating group model was applied to the methyl group. Eleven outliners (-7 13 13, -6 12 12, -9 10 9, -7 1 17, -6 11 16, -9 11 10, -6 11 15, -5 2 16, -7 9 15, -4 0 18, -7 10 15) were omitted in the final refinement.

### Crystal data

**Table:**

C_31_H_22_F_3_NO_2_	*F*(000) = 1032
*M**_r_* = 497.50	*D*_x_ = 1.362 Mg m^−^^3^
Monoclinic, *P*2_1_/*c*	Mo *K*α radiation, λ = 0.71073 Å
Hall symbol: -P 2ybc	Cell parameters from 5213 reflections
*a* = 8.8373 (2) Å	θ = 2.5–30.1°
*b* = 20.1333 (5) Å	µ = 0.10 mm^−^^1^
*c* = 13.7129 (3) Å	*T* = 100 K
β = 96.243 (1)°	Block, yellow
*V* = 2425.39 (10) Å^3^	0.30 × 0.28 × 0.20 mm
*Z* = 4	

### Data collection

**Table:**

Bruker SMART APEXII CCD area-detector diffractometer	7039 independent reflections
Radiation source: fine-focus sealed tube	4753 reflections with *I* > 2σ(*I*)
Graphite monochromator	*R*_int_ = 0.072
φ and ω scans	θ_max_ = 30.1°, θ_min_ = 2.0°
Absorption correction: multi-scan (*SADABS*; Bruker, 2009)	*h* = −10→12
*T*_min_ = 0.971, *T*_max_ = 0.980	*k* = −28→23
27133 measured reflections	*l* = −19→19

### Refinement

**Table:**

Refinement on *F*^2^	Primary atom site location: structure-invariant direct methods
Least-squares matrix: full	Secondary atom site location: difference Fourier map
*R*[*F*^2^ > 2σ(*F*^2^)] = 0.082	Hydrogen site location: inferred from neighbouring sites
*wR*(*F*^2^) = 0.186	H-atom parameters constrained
*S* = 1.10	*w* = 1/[σ^2^(*F*_o_^2^) + (0.062*P*)^2^ + 2.6644*P*] where *P* = (*F*_o_^2^ + 2*F*_c_^2^)/3
7039 reflections	(Δ/σ)_max_ < 0.001
335 parameters	Δρ_max_ = 0.55 e Å^−^^3^
0 restraints	Δρ_min_ = −0.37 e Å^−^^3^

### Special details

**Table:**

Experimental. The crystal was placed in the cold stream of an Oxford Cryosystems Cobra open-flow nitrogen cryostat (Cosier & Glazer, 1986) operating at 100.0 (1) K.
Geometry. All e.s.d.'s (except the e.s.d. in the dihedral angle between two l.s. planes) are estimated using the full covariance matrix. The cell e.s.d.'s are taken into account individually in the estimation of e.s.d.'s in distances, angles and torsion angles; correlations between e.s.d.'s in cell parameters are only used when they are defined by crystal symmetry. An approximate (isotropic) treatment of cell e.s.d.'s is used for estimating e.s.d.'s involving l.s. planes.
Refinement. Refinement of *F*^2^ against ALL reflections. The weighted *R*-factor *wR* and goodness of fit *S* are based on *F*^2^, conventional *R*-factors *R* are based on *F*, with *F* set to zero for negative *F*^2^. The threshold expression of *F*^2^ > σ(*F*^2^) is used only for calculating *R*-factors(gt) *etc*. and is not relevant to the choice of reflections for refinement. *R*-factors based on *F*^2^ are statistically about twice as large as those based on *F*, and *R*- factors based on ALL data will be even larger.

### Fractional atomic coordinates and isotropic or equivalent isotropic displacement parameters (Å^2^)

**Table:**

	*x*	*y*	*z*	*U*_iso_*/*U*_eq_	
F1	0.0010 (2)	0.42817 (7)	0.71415 (11)	0.0310 (4)	
F2	0.0624 (2)	0.49747 (9)	0.82994 (11)	0.0382 (5)	
F3	−0.1474 (2)	0.50882 (9)	0.73716 (14)	0.0416 (5)	
O1	0.6473 (2)	0.62588 (9)	0.59831 (12)	0.0197 (4)	
O2	0.2586 (2)	0.76005 (9)	0.33429 (12)	0.0218 (4)	
N1	0.5391 (2)	0.65334 (10)	0.38454 (14)	0.0159 (4)	
C1	0.6585 (3)	0.67919 (12)	0.55815 (16)	0.0149 (5)	
C2	0.7746 (3)	0.73053 (12)	0.58287 (16)	0.0159 (5)	
C3	0.8914 (3)	0.73588 (13)	0.65826 (17)	0.0189 (5)	
H3A	0.9055	0.7031	0.7083	0.023*	
C4	0.9887 (3)	0.79137 (13)	0.65863 (18)	0.0220 (6)	
H4A	1.0681	0.7963	0.7107	0.026*	
C5	0.9720 (3)	0.83884 (13)	0.58523 (17)	0.0206 (5)	
H5A	1.0404	0.8754	0.5877	0.025*	
C6	0.8547 (3)	0.83393 (12)	0.50641 (17)	0.0181 (5)	
C7	0.8279 (3)	0.87667 (13)	0.42369 (18)	0.0218 (5)	
H7A	0.8897	0.9149	0.4186	0.026*	
C8	0.7125 (3)	0.86261 (13)	0.35127 (17)	0.0212 (5)	
H8A	0.6971	0.8913	0.2961	0.025*	
C9	0.6154 (3)	0.80682 (13)	0.35583 (16)	0.0196 (5)	
H9A	0.5375	0.7981	0.3040	0.024*	
C10	0.6352 (3)	0.76561 (12)	0.43570 (16)	0.0151 (5)	
C11	0.7565 (3)	0.77944 (12)	0.50919 (16)	0.0162 (5)	
C12	0.5532 (3)	0.70278 (11)	0.46338 (15)	0.0146 (5)	
C13	0.3835 (3)	0.71004 (12)	0.48642 (16)	0.0151 (5)	
C14	0.3684 (3)	0.73745 (12)	0.59096 (16)	0.0162 (5)	
H14A	0.2760	0.7198	0.6167	0.019*	
H14B	0.4586	0.7256	0.6369	0.019*	
C15	0.3574 (3)	0.81157 (12)	0.57692 (16)	0.0171 (5)	
C16	0.3775 (3)	0.86177 (13)	0.64670 (18)	0.0201 (5)	
H16A	0.4036	0.8516	0.7141	0.024*	
C17	0.3584 (3)	0.92734 (13)	0.61549 (19)	0.0233 (6)	
H17A	0.3737	0.9622	0.6622	0.028*	
C18	0.3172 (3)	0.94303 (13)	0.5170 (2)	0.0264 (6)	
H18A	0.3039	0.9881	0.4976	0.032*	
C19	0.2956 (3)	0.89312 (13)	0.44737 (18)	0.0222 (5)	
H19A	0.2663	0.9032	0.3803	0.027*	
C20	0.3181 (3)	0.82781 (12)	0.47853 (16)	0.0164 (5)	
C21	0.3106 (3)	0.76631 (12)	0.41957 (16)	0.0159 (5)	
C22	0.3086 (3)	0.64178 (12)	0.45501 (16)	0.0165 (5)	
H22A	0.2291	0.6511	0.3991	0.020*	
C23	0.4350 (3)	0.60207 (12)	0.41273 (17)	0.0182 (5)	
H23A	0.4869	0.5719	0.4627	0.022*	
H23B	0.3934	0.5756	0.3551	0.022*	
C24	0.2298 (3)	0.60506 (12)	0.53182 (17)	0.0167 (5)	
C25	0.0724 (3)	0.60120 (13)	0.52232 (18)	0.0197 (5)	
H25A	0.0153	0.6231	0.4690	0.024*	
C26	−0.0042 (3)	0.56580 (13)	0.58934 (19)	0.0225 (5)	
H26A	−0.1121	0.5631	0.5812	0.027*	
C27	0.0790 (3)	0.53456 (12)	0.66806 (17)	0.0187 (5)	
C28	0.2365 (3)	0.53925 (12)	0.68008 (17)	0.0199 (5)	
H28A	0.2929	0.5187	0.7349	0.024*	
C29	0.3123 (3)	0.57388 (12)	0.61240 (17)	0.0198 (5)	
H29A	0.4202	0.5765	0.6207	0.024*	
C30	0.6816 (3)	0.62840 (13)	0.35438 (18)	0.0225 (6)	
H30A	0.6606	0.6025	0.2940	0.034*	
H30B	0.7321	0.6001	0.4063	0.034*	
H30C	0.7481	0.6659	0.3426	0.034*	
C31	−0.0011 (3)	0.49303 (13)	0.73694 (18)	0.0217 (5)	

### Atomic displacement parameters (Å^2^)

**Table:**

	*U*^11^	*U*^22^	*U*^33^	*U*^12^	*U*^13^	*U*^23^
F1	0.0516 (11)	0.0162 (8)	0.0285 (8)	−0.0061 (7)	0.0187 (7)	−0.0013 (6)
F2	0.0565 (12)	0.0422 (10)	0.0176 (7)	−0.0214 (9)	0.0121 (7)	−0.0018 (7)
F3	0.0346 (10)	0.0408 (11)	0.0548 (11)	0.0048 (8)	0.0291 (9)	0.0143 (9)
O1	0.0204 (9)	0.0198 (9)	0.0192 (8)	−0.0005 (7)	0.0044 (7)	0.0053 (7)
O2	0.0281 (10)	0.0237 (9)	0.0130 (7)	−0.0003 (8)	0.0002 (7)	0.0007 (7)
N1	0.0189 (11)	0.0157 (10)	0.0141 (9)	−0.0004 (8)	0.0060 (8)	−0.0035 (7)
C1	0.0160 (11)	0.0183 (12)	0.0115 (10)	0.0010 (9)	0.0068 (8)	−0.0005 (9)
C2	0.0178 (12)	0.0172 (11)	0.0136 (10)	0.0009 (10)	0.0060 (9)	−0.0023 (9)
C3	0.0195 (12)	0.0242 (13)	0.0139 (10)	0.0012 (10)	0.0053 (9)	−0.0015 (9)
C4	0.0187 (13)	0.0274 (14)	0.0197 (11)	−0.0005 (11)	0.0014 (9)	−0.0060 (10)
C5	0.0190 (13)	0.0225 (13)	0.0212 (12)	−0.0035 (10)	0.0055 (9)	−0.0054 (10)
C6	0.0200 (12)	0.0194 (12)	0.0162 (11)	−0.0006 (10)	0.0082 (9)	−0.0038 (9)
C7	0.0267 (14)	0.0195 (12)	0.0206 (12)	−0.0056 (11)	0.0094 (10)	−0.0012 (10)
C8	0.0282 (14)	0.0228 (13)	0.0142 (11)	−0.0025 (11)	0.0090 (10)	0.0026 (10)
C9	0.0237 (13)	0.0241 (13)	0.0118 (10)	−0.0001 (11)	0.0053 (9)	0.0001 (9)
C10	0.0163 (11)	0.0175 (11)	0.0125 (10)	0.0012 (9)	0.0067 (8)	−0.0023 (9)
C11	0.0179 (12)	0.0194 (12)	0.0121 (10)	0.0021 (10)	0.0051 (8)	−0.0023 (9)
C12	0.0172 (12)	0.0157 (11)	0.0111 (9)	−0.0002 (9)	0.0025 (8)	0.0008 (8)
C13	0.0182 (12)	0.0157 (11)	0.0118 (10)	0.0012 (9)	0.0033 (8)	0.0004 (8)
C14	0.0211 (12)	0.0165 (11)	0.0116 (10)	0.0007 (10)	0.0053 (8)	0.0005 (9)
C15	0.0162 (12)	0.0215 (12)	0.0142 (10)	0.0018 (10)	0.0051 (9)	−0.0015 (9)
C16	0.0202 (13)	0.0236 (13)	0.0174 (11)	0.0003 (10)	0.0067 (9)	−0.0021 (10)
C17	0.0272 (14)	0.0216 (13)	0.0228 (12)	−0.0023 (11)	0.0105 (10)	−0.0073 (10)
C18	0.0336 (16)	0.0173 (13)	0.0298 (13)	0.0024 (11)	0.0093 (11)	0.0036 (10)
C19	0.0263 (14)	0.0213 (13)	0.0200 (11)	0.0032 (11)	0.0072 (10)	0.0036 (10)
C20	0.0176 (12)	0.0171 (11)	0.0154 (10)	0.0010 (10)	0.0050 (9)	0.0012 (9)
C21	0.0158 (11)	0.0188 (12)	0.0137 (10)	0.0001 (10)	0.0045 (8)	0.0027 (9)
C22	0.0179 (12)	0.0166 (11)	0.0155 (10)	−0.0015 (9)	0.0035 (9)	0.0004 (9)
C23	0.0229 (13)	0.0168 (12)	0.0156 (10)	−0.0028 (10)	0.0056 (9)	−0.0019 (9)
C24	0.0216 (13)	0.0134 (11)	0.0162 (10)	−0.0021 (10)	0.0076 (9)	−0.0008 (9)
C25	0.0204 (13)	0.0196 (12)	0.0198 (11)	0.0015 (10)	0.0056 (9)	0.0000 (9)
C26	0.0182 (13)	0.0227 (13)	0.0280 (13)	0.0002 (11)	0.0082 (10)	0.0002 (10)
C27	0.0257 (13)	0.0157 (11)	0.0170 (11)	−0.0013 (10)	0.0129 (9)	−0.0027 (9)
C28	0.0259 (14)	0.0183 (12)	0.0163 (11)	−0.0009 (10)	0.0058 (9)	0.0012 (9)
C29	0.0192 (13)	0.0205 (12)	0.0203 (11)	−0.0008 (10)	0.0049 (9)	0.0001 (10)
C30	0.0231 (13)	0.0237 (13)	0.0217 (12)	0.0012 (11)	0.0069 (10)	−0.0045 (10)
C31	0.0250 (14)	0.0202 (13)	0.0225 (12)	−0.0011 (11)	0.0144 (10)	−0.0019 (10)

### Geometric parameters (Å, º)

**Table:**

F1—C31	1.343 (3)	C14—H14A	0.9900
F2—C31	1.339 (3)	C14—H14B	0.9900
F3—C31	1.332 (3)	C15—C16	1.390 (3)
O1—C1	1.215 (3)	C15—C20	1.395 (3)
O2—C21	1.216 (3)	C16—C17	1.392 (4)
N1—C30	1.457 (3)	C16—H16A	0.9500
N1—C23	1.462 (3)	C17—C18	1.397 (4)
N1—C12	1.465 (3)	C17—H17A	0.9500
C1—C2	1.470 (3)	C18—C19	1.385 (4)
C1—C12	1.586 (3)	C18—H18A	0.9500
C2—C3	1.383 (3)	C19—C20	1.390 (3)
C2—C11	1.408 (3)	C19—H19A	0.9500
C3—C4	1.410 (4)	C20—C21	1.476 (3)
C3—H3A	0.9500	C22—C24	1.517 (3)
C4—C5	1.384 (4)	C22—C23	1.538 (4)
C4—H4A	0.9500	C22—H22A	1.0000
C5—C6	1.417 (3)	C23—H23A	0.9900
C5—H5A	0.9500	C23—H23B	0.9900
C6—C11	1.402 (3)	C24—C25	1.385 (4)
C6—C7	1.423 (3)	C24—C29	1.404 (3)
C7—C8	1.374 (3)	C25—C26	1.395 (4)
C7—H7A	0.9500	C25—H25A	0.9500
C8—C9	1.419 (4)	C26—C27	1.389 (3)
C8—H8A	0.9500	C26—H26A	0.9500
C9—C10	1.370 (3)	C27—C28	1.387 (4)
C9—H9A	0.9500	C27—C31	1.495 (4)
C10—C11	1.417 (3)	C28—C29	1.390 (4)
C10—C12	1.526 (3)	C28—H28A	0.9500
C12—C13	1.573 (4)	C29—H29A	0.9500
C13—C21	1.553 (3)	C30—H30A	0.9800
C13—C14	1.555 (3)	C30—H30B	0.9800
C13—C22	1.565 (3)	C30—H30C	0.9800
C14—C15	1.506 (3)		
			
C30—N1—C23	114.8 (2)	C17—C16—H16A	120.8
C30—N1—C12	115.90 (19)	C16—C17—C18	121.5 (2)
C23—N1—C12	106.87 (18)	C16—C17—H17A	119.3
O1—C1—C2	127.3 (2)	C18—C17—H17A	119.3
O1—C1—C12	124.5 (2)	C19—C18—C17	120.3 (2)
C2—C1—C12	108.11 (19)	C19—C18—H18A	119.9
C3—C2—C11	120.0 (2)	C17—C18—H18A	119.9
C3—C2—C1	132.3 (2)	C18—C19—C20	118.1 (2)
C11—C2—C1	107.72 (19)	C18—C19—H19A	121.0
C2—C3—C4	118.0 (2)	C20—C19—H19A	121.0
C2—C3—H3A	121.0	C19—C20—C15	122.1 (2)
C4—C3—H3A	121.0	C19—C20—C21	128.9 (2)
C5—C4—C3	121.9 (2)	C15—C20—C21	109.0 (2)
C5—C4—H4A	119.1	O2—C21—C20	127.1 (2)
C3—C4—H4A	119.1	O2—C21—C13	125.6 (2)
C4—C5—C6	121.2 (2)	C20—C21—C13	107.31 (18)
C4—C5—H5A	119.4	C24—C22—C23	114.6 (2)
C6—C5—H5A	119.4	C24—C22—C13	116.69 (19)
C11—C6—C5	115.9 (2)	C23—C22—C13	104.93 (19)
C11—C6—C7	116.4 (2)	C24—C22—H22A	106.7
C5—C6—C7	127.6 (2)	C23—C22—H22A	106.7
C8—C7—C6	119.9 (2)	C13—C22—H22A	106.7
C8—C7—H7A	120.1	N1—C23—C22	103.67 (19)
C6—C7—H7A	120.1	N1—C23—H23A	111.0
C7—C8—C9	122.4 (2)	C22—C23—H23A	111.0
C7—C8—H8A	118.8	N1—C23—H23B	111.0
C9—C8—H8A	118.8	C22—C23—H23B	111.0
C10—C9—C8	119.3 (2)	H23A—C23—H23B	109.0
C10—C9—H9A	120.3	C25—C24—C29	118.6 (2)
C8—C9—H9A	120.4	C25—C24—C22	119.6 (2)
C9—C10—C11	118.1 (2)	C29—C24—C22	121.7 (2)
C9—C10—C12	132.7 (2)	C24—C25—C26	121.4 (2)
C11—C10—C12	109.21 (19)	C24—C25—H25A	119.3
C6—C11—C2	123.0 (2)	C26—C25—H25A	119.3
C6—C11—C10	123.9 (2)	C27—C26—C25	119.4 (2)
C2—C11—C10	113.0 (2)	C27—C26—H26A	120.3
N1—C12—C10	112.60 (19)	C25—C26—H26A	120.3
N1—C12—C13	101.81 (18)	C28—C27—C26	120.0 (2)
C10—C12—C13	117.51 (19)	C28—C27—C31	120.0 (2)
N1—C12—C1	113.36 (18)	C26—C27—C31	119.9 (2)
C10—C12—C1	101.53 (17)	C27—C28—C29	120.3 (2)
C13—C12—C1	110.51 (18)	C27—C28—H28A	119.8
C21—C13—C14	102.34 (18)	C29—C28—H28A	119.8
C21—C13—C22	110.03 (17)	C28—C29—C24	120.2 (2)
C14—C13—C22	119.3 (2)	C28—C29—H29A	119.9
C21—C13—C12	107.02 (19)	C24—C29—H29A	119.9
C14—C13—C12	113.39 (18)	N1—C30—H30A	109.5
C22—C13—C12	104.30 (19)	N1—C30—H30B	109.5
C15—C14—C13	104.15 (18)	H30A—C30—H30B	109.5
C15—C14—H14A	110.9	N1—C30—H30C	109.5
C13—C14—H14A	110.9	H30A—C30—H30C	109.5
C15—C14—H14B	110.9	H30B—C30—H30C	109.5
C13—C14—H14B	110.9	F3—C31—F2	106.7 (2)
H14A—C14—H14B	108.9	F3—C31—F1	105.7 (2)
C16—C15—C20	119.7 (2)	F2—C31—F1	105.8 (2)
C16—C15—C14	129.1 (2)	F3—C31—C27	113.2 (2)
C20—C15—C14	111.2 (2)	F2—C31—C27	112.7 (2)
C15—C16—C17	118.4 (2)	F1—C31—C27	112.2 (2)
C15—C16—H16A	120.8		
			
O1—C1—C2—C3	−5.3 (5)	C22—C13—C14—C15	−145.3 (2)
C12—C1—C2—C3	178.6 (3)	C12—C13—C14—C15	91.2 (2)
O1—C1—C2—C11	171.9 (2)	C13—C14—C15—C16	−163.4 (3)
C12—C1—C2—C11	−4.2 (3)	C13—C14—C15—C20	18.0 (3)
C11—C2—C3—C4	0.4 (4)	C20—C15—C16—C17	−0.4 (4)
C1—C2—C3—C4	177.3 (3)	C14—C15—C16—C17	−178.8 (3)
C2—C3—C4—C5	−1.4 (4)	C15—C16—C17—C18	1.2 (4)
C3—C4—C5—C6	0.5 (4)	C16—C17—C18—C19	−0.6 (4)
C4—C5—C6—C11	1.4 (4)	C17—C18—C19—C20	−0.8 (4)
C4—C5—C6—C7	−176.6 (3)	C18—C19—C20—C15	1.7 (4)
C11—C6—C7—C8	−1.2 (4)	C18—C19—C20—C21	−176.6 (3)
C5—C6—C7—C8	176.8 (3)	C16—C15—C20—C19	−1.1 (4)
C6—C7—C8—C9	0.9 (4)	C14—C15—C20—C19	177.6 (2)
C7—C8—C9—C10	0.9 (4)	C16—C15—C20—C21	177.5 (2)
C8—C9—C10—C11	−2.2 (4)	C14—C15—C20—C21	−3.8 (3)
C8—C9—C10—C12	−179.9 (3)	C19—C20—C21—O2	−13.3 (4)
C5—C6—C11—C2	−2.5 (4)	C15—C20—C21—O2	168.3 (3)
C7—C6—C11—C2	175.8 (2)	C19—C20—C21—C13	166.2 (3)
C5—C6—C11—C10	−178.5 (2)	C15—C20—C21—C13	−12.3 (3)
C7—C6—C11—C10	−0.2 (4)	C14—C13—C21—O2	−158.2 (2)
C3—C2—C11—C6	1.6 (4)	C22—C13—C21—O2	−30.4 (3)
C1—C2—C11—C6	−176.0 (2)	C12—C13—C21—O2	82.3 (3)
C3—C2—C11—C10	178.0 (2)	C14—C13—C21—C20	22.3 (2)
C1—C2—C11—C10	0.4 (3)	C22—C13—C21—C20	150.1 (2)
C9—C10—C11—C6	1.9 (4)	C12—C13—C21—C20	−97.1 (2)
C12—C10—C11—C6	−179.9 (2)	C21—C13—C22—C24	−120.0 (2)
C9—C10—C11—C2	−174.4 (2)	C14—C13—C22—C24	−2.2 (3)
C12—C10—C11—C2	3.8 (3)	C12—C13—C22—C24	125.5 (2)
C30—N1—C12—C10	61.1 (3)	C21—C13—C22—C23	112.0 (2)
C23—N1—C12—C10	−169.48 (19)	C14—C13—C22—C23	−130.3 (2)
C30—N1—C12—C13	−172.15 (19)	C12—C13—C22—C23	−2.5 (2)
C23—N1—C12—C13	−42.7 (2)	C30—N1—C23—C22	171.83 (18)
C30—N1—C12—C1	−53.5 (3)	C12—N1—C23—C22	41.8 (2)
C23—N1—C12—C1	76.0 (2)	C24—C22—C23—N1	−151.82 (18)
C9—C10—C12—N1	50.4 (4)	C13—C22—C23—N1	−22.5 (2)
C11—C10—C12—N1	−127.4 (2)	C23—C22—C24—C25	−128.6 (2)
C9—C10—C12—C13	−67.4 (3)	C13—C22—C24—C25	108.2 (2)
C11—C10—C12—C13	114.8 (2)	C23—C22—C24—C29	50.6 (3)
C9—C10—C12—C1	172.0 (3)	C13—C22—C24—C29	−72.5 (3)
C11—C10—C12—C1	−5.8 (2)	C29—C24—C25—C26	−1.7 (4)
O1—C1—C12—N1	−49.2 (3)	C22—C24—C25—C26	177.6 (2)
C2—C1—C12—N1	127.1 (2)	C24—C25—C26—C27	0.9 (4)
O1—C1—C12—C10	−170.2 (2)	C25—C26—C27—C28	0.6 (4)
C2—C1—C12—C10	6.0 (2)	C25—C26—C27—C31	−176.3 (2)
O1—C1—C12—C13	64.4 (3)	C26—C27—C28—C29	−1.5 (4)
C2—C1—C12—C13	−119.4 (2)	C31—C27—C28—C29	175.4 (2)
N1—C12—C13—C21	−90.2 (2)	C27—C28—C29—C24	0.7 (4)
C10—C12—C13—C21	33.3 (2)	C25—C24—C29—C28	0.8 (4)
C1—C12—C13—C21	149.07 (18)	C22—C24—C29—C28	−178.4 (2)
N1—C12—C13—C14	157.69 (18)	C28—C27—C31—F3	160.1 (2)
C10—C12—C13—C14	−78.8 (2)	C26—C27—C31—F3	−23.0 (3)
C1—C12—C13—C14	37.0 (3)	C28—C27—C31—F2	38.9 (3)
N1—C12—C13—C22	26.4 (2)	C26—C27—C31—F2	−144.2 (2)
C10—C12—C13—C22	149.85 (18)	C28—C27—C31—F1	−80.4 (3)
C1—C12—C13—C22	−94.3 (2)	C26—C27—C31—F1	96.5 (3)
C21—C13—C14—C15	−23.7 (2)		

### Hydrogen-bond geometry (Å, º)

Cg1 is the centroid of the C15–C20 ring.

**Table:**

*D*—H···*A*	*D*—H	H···*A*	*D*···*A*	*D*—H···*A*
C29—H29*A*···O1	0.95	2.29	3.166 (3)	153
C4—H4*A*···O2^i^	0.95	2.52	3.364 (3)	147
C16—H16*A*···N1^ii^	0.95	2.51	3.429 (3)	163
C26—H26*A*···O1^iii^	0.95	2.51	3.324 (3)	144
C5—H5*A*···*Cg*1^iv^	0.95	2.74	3.417 (3)	129

Symmetry codes: (i) *x*+1, −*y*+3/2, *z*+1/2; (ii) *x*, −*y*+3/2, *z*+1/2; (iii) *x*−1, *y*, *z*; (iv) *x*+1, *y*, *z*.
